# Post-antifungal effect of the combination of anidulafungin with amphotericin B and fluconazole against fluconazole-susceptible and -resistant *Candida albicans*

**DOI:** 10.18502/cmm.8.2.10327

**Published:** 2022-06

**Authors:** Narges Vaseghi, Majid Piramoon, Shaghayegh Khojasteh, Kiana Abbasi, Sahar Mohseni, Javad Javidnia, Behrooz Naghili, Narges Aslani

**Affiliations:** 1 Department of Pathobiology, Science and Research Branch, Islamic Azad University, Tehran, Iran; 2 Department of Medicinal Chemistry and Radiopharmacy, School of Pharmacy, Lorestan University of Medical Sciences, Khorramabad, Iran; 3 Razi Herbal Medicines Research Center, Lorestan University of Medical Sciences, Khorramabad, Iran; 4 Invasive Fungi Research Center, Communicable Diseases Institute, Mazandaran University of Medical Sciences, Sari, Iran; 5 Student Research Committee, Mazandaran University of Medical Sciences, Sari, Iran; 6 Department of Microbiology, Zanjan Branch, Islamic Azad University, Zanjan, Iran; 7 Department of Microbiology, Sari Branch, Islamic Azad University, Sari, Iran; 8 Department of Mycology, School of Medicine, Mazandaran University of Medical Science, Mazandaran, Sari, Iran; 9 Infectious and Tropical Diseases Research Center, Tabriz University of Medical Sciences, Tabriz, Iran

**Keywords:** Anidulafungin, *Candida albicans*, Combination regimen, Post-antifungal effect

## Abstract

**Background and Purpose:**

Invasive candidiasis is a life-threatening condition that kills a large number of immunocompromised patients each year worldwide. We used post-antifungal effect studies to analyze the activities of anidulafungin (AFG), as a clinically crucial antifungal drug, amphotericin B (AMB), and fluconazole (alone and in combinations) against FLC-susceptible and -resistant *Candida albicans* (*C. albicans*) isolates obtained from the cancer patients.

**Materials and Methods:**

We tested the phenomenon of post antifungal effects of FLC, AMB, AFG, and combinations of FLC+AFG, AFG+AMB, and FLC+AMB against 17 *C. albicans* isolates obtained from the oral cavity of cancer patients. Isolates that had not been exposed to antifungals, served as a control group. Colony counts were performed at 0, 2, 4, 6, and 24 h after a brief (1 h) exposure to antifungal.

**Results:**

The FLC had no detectable post-antifungal effect independent of antifungal concentration and resembled drug-free FLC (control). Significant variations in the post-antifungal effect were observed when all AMB and AFG were compared to FLC. The combination of AFG and AMB with FLC resulted in effective activity compared to FLC alone. Combination regimens were rated as indifferent in general. Interestingly, low dosages of the AFG displayed increasing fungistatic action as it approached a fungistatic endpoint against *C. albicans* isolates (n=17).

**Conclusion:**

Our findings suggested that brief exposure to AFG, in combination with FLC and AMB, at low concentrations of the medicines utilized, could be effective in the evaluation and optimization of new dosage regimens to manage candidiasis. However, future studies will determine the clinical utility of our findings.

## Introduction

*Candida* infection (candidiasis) is a life-threatening condition that causes significant morbidity and mortality worldwide. The prevalence of candidiasis has risen considerably in recent years, owing primarily to an increase in the number of immunocompromised people [ 1 ]. Despite a global trend in candidiasis epidemiology toward an increasing prevalence of non-*albicans* *Candida* species, *Candida albicans* remains the most commonly reported pathogenic yeast [ 2 , 3 ]. Fluconazole (FLC) is the preferred antifungal for treating candidiasis owing to low toxicity, wide tissue distribution, and high solubility. However, candidiasis therapy is problematic due to frequent relapses and treatment failures [ 4 ]. One of the most critical factors contributing to the progressive development of azole-resistant fungi appears to be the widespread use of FLC for prophylaxis or pre-emptive treatment [ 5 , 6 ]. Therefore, the combination of antifungal regimens should be narrowed to avoid further emergence of resistance and treatment failure, based on *in vitro* activity and post-antifungal effect (PAFE) profiles. The therapeutic significance of *in vitro* PAFE, in conjunction with a drug’s minimum inhibitory concentration (MIC) data, is linked to evaluating novel dosage regimens for new antifungal medications or combinations of agents *in vivo* during clinical use [ 7 ]. 

To explain the effect of azole-echinocandin and/or polyene combinations, we conducted PAFE studies to evaluate and compare the activities of anidulafungin (AFG) as a clinically important antifungal drug, amphotericin B (AMB), and FLC alone and in combination, against FLC-susceptible and -resistant *C. albicans* isolates derived from the cancer patients.

## Materials and Methods

### Fungal strains

This study was conducted on eight FLC-resistant clinical strains of *C. albicans* and nine FLC-susceptible strains. The isolates were taken from the oral cavity of patients with hematological malignancies and oncological disorders at the cancer center of Mazandaran University Hospital, Sari, Iran. Matrix-assisted laser desorption/ionization time-of-flight mass spectrometry (MALDI-TOF MS) was used to identify all clinical isolates earlier [ 8 ]. These isolates had been stored at -70 °C at the reference culture collection of invasive fungi research center (IFRC, Sari, Iran) in cryo-tubes (Mast Diagnostics, Bootle, Merseyside, UK). The clinical strains were subcultured twice onto Sabouraud dextrose agar (SDA) before usage.

This study was approved by the Ethics Committee of Mazandaran University of Medical Sciences, Sari, Iran (Nr. 1298).

### Antifungal agents and media

Fluconazole (FLC; Pfizer, Groton, CT, USA), amphotericin B (AMB; Sigma, St. Louis, MO, USA), and anidulafungin (AFG; Pfizer SLU, Madrid, Spain) were obtained as reagent-grade powders from the respective manufacturers and used for the preparation of the Clinical and Laboratory Standards Institute (CLSI) microdilution trays. Microplates for each drug were prepared using RPMI 1640 medium (Gibco, UK) containing L-glutamine and lacking sodium bicarbonate, buffered with 3-(N-morpholino)-propanesulfonic acid (MOPS 0.165 M; pH 7.0) (Sigma-Aldrich, Madrid, Spain), dissolved in one liter of sterile distilled water, sterilized with filter, and stored at -70 °C before use. AMB, FLC, and AFG were prepared at the final concentrations of 0.016-16 μg/ml, 0.063-64 μg/ml, and 0.008-8 μg/ml, respectively.

### Determination of minimum inhibitory concentration

After 24 h of incubation at 35 °C, all *Candida* isolates were tested for antifungal susceptibility, according to the CLSI guidelines M27-A3 and M27-S4 documents, as validated recently by Pfaller et al. [ 9 - 12 ]. The MIC endpoint was set at 100% inhibition for AMB and greater than 50% inhibition for the other antifungal drugs. All of the tests were done twice in each round.

### Post-antifungal effect (PAFE)

The post-antifungal effect investigations were carried out as previously stated [ 13 ]. Briefly, the PAFEs were determined for each strain alone or in the combination with three drugs (1×MIC, 4×MIC, 16×MIC of FLC alone; 0.25×MIC, 1×MIC, and 4×MIC of AMB alone; 0.125×MIC, 1×MIC, and 4×MIC of AFG alone; 1×MIC-FLC+1×MIC-AFG; 4×MIC-FLC+4×MIC-AFG; 1×MIC-FLC+1×MIC- AmB; 4×MIC-FLC+4×MIC-AMB; 1×MIC-AFG+1×MIC-AMB; 4×MIC-AFG+4×MIC-AMB. A hemocytometer slide was used to modify the turbidity concentration of yeast cell suspensions in sterile distilled water (1×10^6^ CFU/mL). Afterwards, 1 mL of the yeast suspension was added to 9 mL of RPMI 1640 medium with and without the drug (control). Antifungal agents were removed by three cycles of repeated centrifugation (2000 rpm, 10 min) and washing with sterile PBS after a brief exposure to concentrations of antifungal agents (1 h at 35 °C). The supernatant was decanted entirely after the final centrifugation, and the fungal pellets were suspended in 9 mL warm RPMI. The solutions were incubated with gentle agitation at 35 °C. At the predesigned time points (0, 2, 4, 6, and 24 h), a 100 μL suspension from each solution was serially diluted and a 30 μL aliquot was covered onto an SDA plate for CFU counting. After a 48-hour incubation period at 35 °C, colonies were counted [ 13 ]. For each isolate, PAFE studies were carried out twice. PAFE refers to the time it takes for antifungal drug-treated cells to recover from the drug’s inhibitory effect, as measured by an increase in CFU/ml of culture. The fungicidal activity was defined as a ≥3 log10 (99.9%) reduction in CFU/ml from the starting inoculum size, and the fungistatic activity was defined as a <99.9% reduction in CFU/mL from the starting inoculum size [ 14 ]. Synergy was defined as a ≥2 log10 increase in the killing activity of the combination. In contrast, antagonism was defined as a ≥2 log10 decrease in killing activity of combinations compared to the most active medication alone at the same concentration.

The interaction would be classified as indifferent if the variation was less than 100-fold [ 15 ]. Plots showing averaged colony counts (log10 CFU/ml) over time were created and compared to a drug-free control (control).

### Statistical analysis

The SPSS software (version 16.0) was used to analyze the data. T-test was used to examine the changes in PAFE following the exposure to various amounts of the three antifungals and their combinations.

*P-value* less than 0.001 (*P*<0.001) was considered statistically significant.

## Results

### Minimal inhibitory concentration

Table 1 summarizes the *in vitro* antifungal susceptibility results. Isolates had MIC ranges of 0.008 to 0.25 μg/ml for AFG, 0.063 to 64.0 μg/ml for FLC, and 0.031 to 16 μg/ml for AMB. In total, eight *C. albicans* isolates were resistant to FLC (MIC ≥8 μg/ml), whereas the remaining nine isolates were susceptible to FLC (MIC range of 0.063 - 4 μg/ml). All *C. albicans* isolates were susceptible to AFG. In addition, eight *C. albicans* isolates were resistant to AMB (MIC ≥2 μg/ml).

**Table 1:** *In vitro* antifungal susceptibilities of 17 clinical *Candida albicans* isolates to three antifungal agents.

*Candida albicans* isolates code (n=17)	Antifungal drugs MIC (µg/mL)		
	Amphotericin B	Fluconazole	Anidulafungin
1308	2	64*	0.008
1327	0.25	64	0.063
1322	1	64	0.125
1333(b)	1	64	0.063
1386 (a)	4	16	0.008
1421(a)	2	16	0.125
1421(b)	0.25	8	0.063
1351	0.125	8	0.063
1309	4	1	0.016
1311	4	4	0.031
1315	0.5	4	0.016
1319	4	0.063	0.063
1320	4	2	0.063
1334	1	2	0.25
1373	16	1	0.016
1381	0.031	0.063	0.008
1392 (a)	0.031	0.125	0.016

### Post-antifungal effect

**Figure 1:**
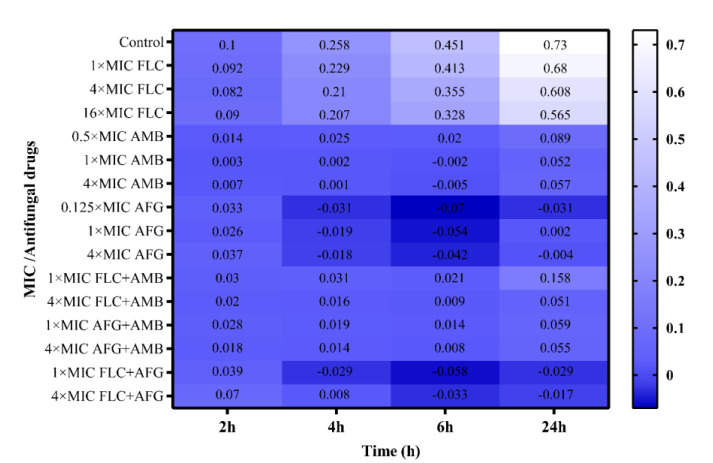
Log of each drug in log CFU/mL compared to starting inoculum size in post-antifungal (PAFE) studies in fluconazole-resistant *Candida albicans* isolates

**Figure 2:**
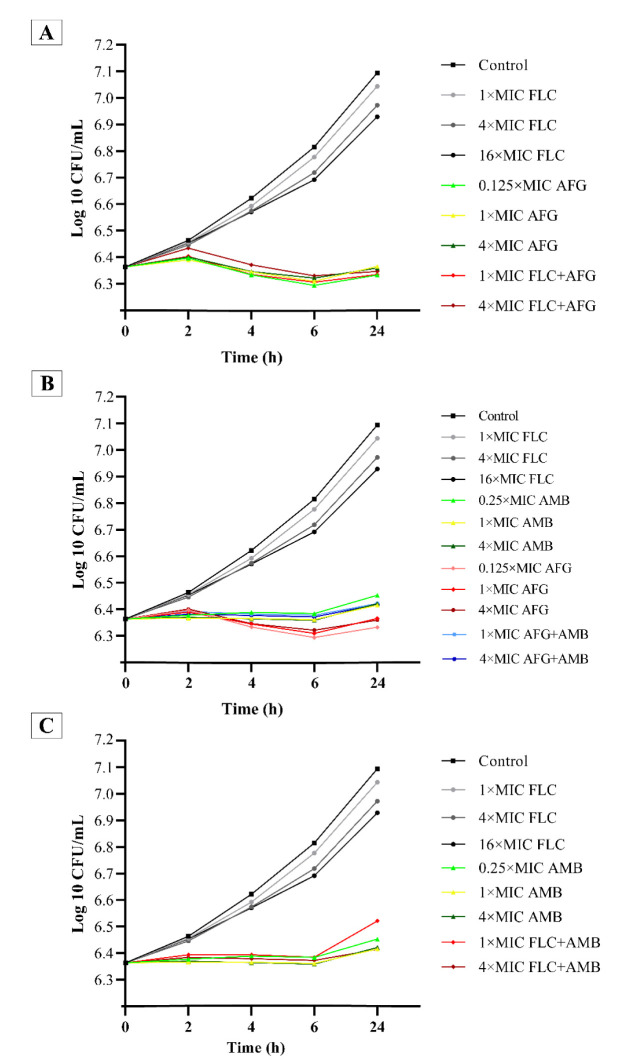
Mean post-antifungal effect curves of FLC, AMB, AFG, and their combinations against eight clinical fluconazole-resistant *Candida albicans* isolates

**Figure 3:**
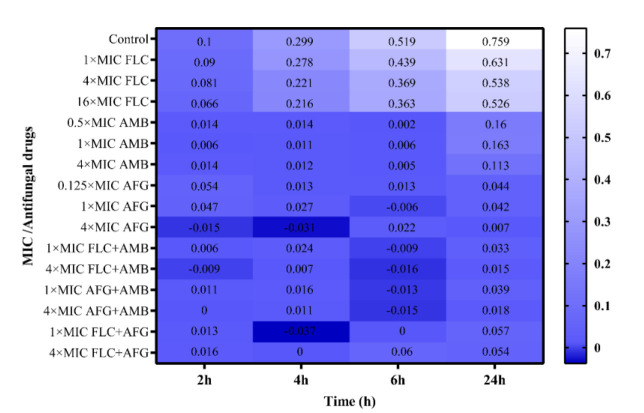
Log of each drug in log CFU/mL compared to starting inoculum size in post-antifungal (PAFE) studies in fluconazole-susceptible *Candida albicans* isolates

**Figure 4:**
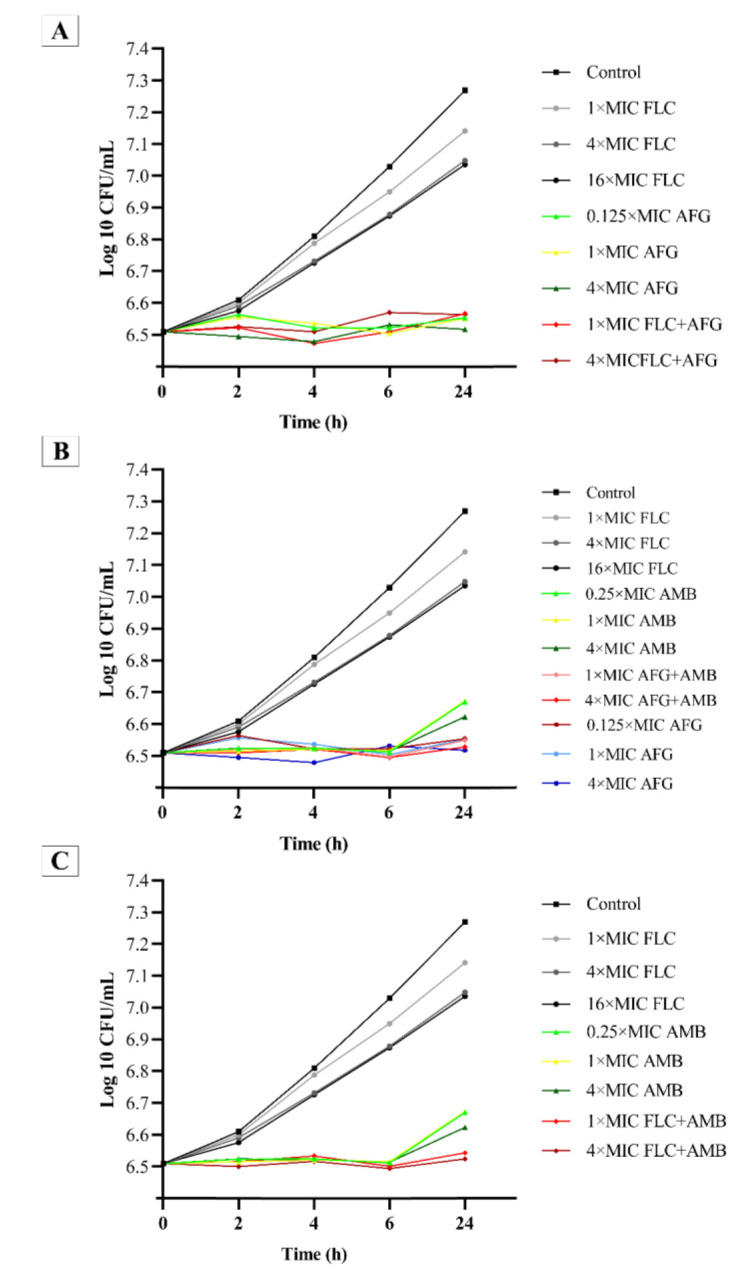
Mean post-antifungal effect curves of FLC, AMB, AFG, and their combinations against nine clinical fluconazole–susceptible *Candida albicans* isolates

Figures 1-4 represent the post-antifungal effects of the three antifungal medications and their combination on 17 FLC-susceptible and -resistant *C. albicans* isolates after 1 h of exposure to and removal of the medicines. Regardless of antifungal concentration, the FLC did not show any significant PAFE (with curves similar to drug-free for FLC-susceptible and -resistant *C. albicans* isolates) (*P*≥0.001). All AFG concentrations and AFG+FLC regimens showed fungistatic efficacy (*P*<0.001) against the FLC-resistant *C. albicans* isolates (n=8) (Figure 1, Figure 2A). Furthermore, there was some fungistatic activity at doses of one and four times the MICs of AMB alone (significantly at 6 h) (Figure 1). 

At all doses, the PAFEs of AMB with FLC combinations generated neither fungicidal nor fungistatic activity (*P*≥0.001) (Fig 1, Fig 2C). At all concentrations, AFG appeared to be slightly more effective than AMB alone, as well as in combination with AFG at one and four times the concentration of AMB (Fig 2B). The AMB-combined regimens did not improve the rate or degree of activity supplied by AFG; therefore, they were classed as indifferent (Figure 2B). 

Furthermore, we discovered that AFG with FLC combination was the most effective drug at 1 × MIC (significant within the first 6 h) (*P*<0.001) (Figure 1).

FLC-susceptible *C. albicans* isolates (n=9) showed fungistatic activity at one time the MIC of AFG+FLC, one and four times the MICs of AFG, and one and four times the MICs of AMB+FLC, respectively (Figure 3, Figure 4). AFG at 4×MIC and in combination with FLC at 1×MIC both produced similar results and were the most effective concentrations (*P*<0.001) (Figure 3). For FLC-susceptible *C. albicans* isolates, the addition of AMB at one and four times the concentrations increased FLC activity (Figure 4C).

When the medications were examined separately and in combination, FLC-susceptible and -resistant *C. albicans* isolates showed substantially identical patterns. When compared to FLC-susceptible *C. albicans* isolates, the combination of AFG with FLC and AFG alone appeared to be marginally more active against FLC-resistant *C. albicans* isolates. For all isolates, AMB and AFG did not show significant dose-dependent PAFE. When comparing FLC alone and control with all concentrations of AMB and AFG, substantial variations in PAFE were identified. AFG reached a fungistatic endpoint at all doses against *C. albicans* isolates (n=17), although interestingly low concentrations displayed higher fungistatic activity. The combination of AFG and FLC demonstrated effective action when compared to FLC alone.

## Discussion

Although the *Candida* genus has over 150 species, *C. albicans* is the most prevalent cause of candidiasis isolated from clinical samples. Severe candidiasis has been much more common in recent years, owing to a large population of high-risk persons who utilized chemotherapeutic, immunosuppressive, and broad-spectrum antifungal medications [ 1 , 16 ]. The long-term use of FLC, as the first-line therapy for prophylaxis and treatment in immunocompromised patients, has been linked to the development of drug resistance in *Candida* species [ 17 ]. Therefore, innovative therapeutic techniques, such as combination medications, may be a viable option for enhancing clinical outcomes, increasing efficacy, and lowering antifungal toxicity. *In vitro* evaluation of the efficiency of these combinations could help researchers find the most effective, powerful, and safe antifungal agents for treating severe infectious diseases.

When comparing all concentrations of AMB and AFG to the control group, we found significant changes in PAFE, whereas FLC did not produce any measurable PAFE. The low growth inhibitory action of FLC against *C. albicans* isolates *in vitro* is one rationale for the absence of significant PAFE after exposure to FLC, as established in many prior investigations [ 18 - 20 ]. As previously stated, echinocandins do not contain ergosterol; therefore, these antifungals should not cause antagonism in combination with azole drugs, such as FLC. At one and four times the MIC, the combination of AFG with FLC showed improved activity and resulted in fungistatic activity with no antagonistic interaction. However, the combination of AFG with AMB was not superior to AFG alone and was classed as indifferent. The concentrations employed in studies mentioning the fungicidal activity of AMB and AFG against *C. albicans* isolates varied significantly from the ones examined in the current study [ 21 , 22 ]. The PAFE phenomenon, on the other hand, is highly dependent on the fungus species, antifungal drug class, inoculum size, drug concentration, research methodology, and drug exposure time [ 23 ].

The PAFE phenomenon, on the other hand, is highly dependent on the fungus species, antifungal drug class, inoculum size, drug concentration, research methodology, and drug exposure time [ 21 ].

Although the PAFEs of AFG, FLC, and AMB against various *Candida* species have been evaluated in a few studies, to our knowledge, limited data is comparing the PAFEs of these antifungals and their combinations against fluconazole-susceptible and -resistant *Candida albicans* isolated from the oral cavity of cancer patients [ 7 , 18 , 20 , 22 ]. AMB showed a prolonged PAFE of more than 12 h against *C. albicans* in the first study examining the PAFE of echinocandins (caspofungin) when evaluated at concentrations ranging from 0.125 to 4 times the MICs [ 13 ]. PAFEs of AFG, FLC, and AMB against clinical isolates of *C. glabrata*, *C. guilliermondii*, *C. krusei*, *C. tropicalis*, and *C. parapsilosis* were determined in another investigation. FLC displayed no measurable PAFE regardless of the concentration, and AFG revealed fungicidal activity against *C. krusei*, *C. glabrata*, and *C. parapsilosis* at four and 16 times the MICs, and AMB elicited a consistently high PAFE in *C. tropicalis* [ 20 , 22 ]. Ellepola [ 21 ] found that the mean duration of AMB-induced PAFE was lowest for *Candida albicans* and highest for *Candida parapsilosis*, with intermediate values for *C. guilliermondii*, *C. glabrata*, *C. krusei*, and *C. tropicalis*. AMB also had the longest PAFE against *C. albicans*, *C. krusei*, and *C. glabrata*, which were all dependent on antifungal drug concentrations and exposure periods [ 24 ]. In PAFE tests, Nguyen et al. [ 22 ] discovered that 1 h exposure of *C. albicans*, *C. glabrata*, *C. parapsilosis*, and *C. krusei* isolates to AFG, at four and 16 times the MICs, resulted in fungicidal levels for >12h, following the drug washout. Furthermore, Gil-Alonso et al. [ 25 ] previously demonstrated that micafungin produced extended PAFE (37.5 h) against all *C. albicans* strains at two times the MICs. It has been shown previously that FLC produced a significant decrease against *C. albicans* isolates; however, researchers have demonstrated that fluconazole showed no measurable PAFE, regardless of the tested concentration, which was consistent with the results obtained in the current study [ 13 , 18 - 20 ]. Our findings suggested that AMB and AFG had a fungistatic activity, somewhat independent of concentration, whereas FLC did not elicit significant PAFE at any of the concentrations tested with all *C. albicans* isolates. AFG and AMB have been described as exhibiting fungistatic activity, independent of the PAFEs concentration. Although fungistatic, FLC did not produce any measurable PAFE. Antifungal combinations of AFG, FLC, and AMB have demonstrated encouraging efficacy against a variety of fungal isolates (*Candida* species, *Cryptococcus neoformans*, and *Aspergillus* species), with no evidence of antagonism [ 15 , 26 ]. Interestingly, in fluconazole-susceptible and -resistant *C. albicans*, the combination of AFG and FLC was superior to FLC alone (at all concentrations), whereas AFG alone was superior to the combination of AFG and FLC in all isolates. There were no notable changes when AFG and AMB were combined. In fact, the combination of AFG and AMB resulted in general apathy. In fluconazole-resistant *Candida albicans*, AFG and AMB alone outperformed the combination of AFG and AMB. Since the effectiveness of medicine on *C. albicans* isolates is detected at low concentrations, PAFEs derived from the combination of AFG and FLC may have clinical significance.

## Conclusion

To our knowledge, this is the first investigation of PAFE created by the combination of these three medications on fluconazole-susceptible and -resistant *Candida albicans* isolated from oral cavities of cancer patients. Surprisingly, AFG alone, was more efficacious on fluconazole-resistant *C. albicans* isolates at lower compared to higher concentrations. Overall, PAFE was not dose-dependent when AFG and FLC were used together. In contrast, the azole antifungal FLC does not create a detectable PAFE against all isolates. Additional in vivo research is required to corroborate these in vitro observations. Eventually, PAFE results, together with MIC values, would be beneficial in determining the best dose regimens in the treatment of *Candida* infections in the clinic.

## Acknowledgments

This study was supported by the School of Medicine, Mazandaran University of Medical Sciences, Sari, Iran (grant no. 1298).

## Authors’ contribution

N. A. contributed to the study design, data analysis, and interpretation, approval of the final version of the manuscript to be published. M. P. and N. V. prepared the strains. S. K., K. A., S. M., B. N., and J. J. performed the experiments. All authors read and approved the final manuscript. 

## Conflicts of interest

The authors declare that they have no competing interests to express.

## Financial disclosure

No financial interests have been declared.
